# Incorporation of Nanomaterials in Glass Ionomer Cements—Recent Developments and Future Perspectives: A Narrative Review

**DOI:** 10.3390/nano12213827

**Published:** 2022-10-29

**Authors:** Radu Claudiu Fierascu

**Affiliations:** 1 National Institute for Research & Development in Chemistry and Petrochemistry—ICECHIM Bucharest, 202 Spl. Independentei, 060021 Bucharest, Romania; fierascu.radu@icechim.ro; 2 Faculty of Chemical Engineering and Biotechnologies, University “Politehnica” of Bucharest, Bucharest, 313 Splaiul Independentei Str., 060042 Bucharest, Romania

**Keywords:** glass ionomer cements, dental caries, periodontal diseases, nanoparticles, apatite, nanostructures

## Abstract

Glass ionomer cements (GICs), restorative materials with commercial availability spanning over five decades, are widely applied due to their advantages (including bio-compatibility, fluoride release, or excellent bonding properties). However, GICs have shortcomings. Among the disadvantages limiting the application of GICs, the poor mechanical properties are the most significant. In order to enhance the mechanical or antimicrobial properties of these materials, the addition of nanomaterials represents a viable approach. The present paper aims to review the literature on the application of different types of nanomaterials for the enhancement of GICs’ mechanical and antimicrobial properties, which could lead to several clinical benefits, including better physical properties and the prevention of tooth decay. After applying the described methodology, representative articles published in the time period 2011-present were selected and included in the final review, covering the modification of GICs with metallic nanoparticles (Cu, Ag), metallic and metalloid oxide nanoparticles (TiO_2_, ZnO, MgO, Al_2_O_3_, ZrO_2_, SiO_2_), apatitic nanomaterials, and other nanomaterials or multi-component nanocomposites.

## 1. Introduction

As recently proposed, the general concept of health is represented by the ability of each individual to adapt to various physiological variations, also known as allostasis [1]. Probably one of the most dynamic in the entire body, the allostasis of the oral cavity represents a very complex phenomenon, with the balance of the species affected by multiple physiological or hormonal factors [2]. Two of the most encountered oral diseases resulting in the imbalance of this equilibrium, caries and periodontal disease, are commonly encountered in the general population—especially in that of industrialized countries—with important social and economic impacts [3,4,5]. One of the main factors responsible for the appearance of dental caries is the acidic attack of cariogenic bacteria (which are commonly found in dental plaque biofilm growth). The tooth decay represents a biofilm-dependent infectious disease, and its main action route is the demineralization of the tooth [5].

Given the complexity of oral biofilms, the development of effective novel dental materials is often difficult. As the oral cavity is an ideal microbial growth environment, any imbalance of microbial community members present at any time could lead to the development of chronic pathological conditions (such as gingivitis and periodontitis), which can further lead to a wide range of complications [6].

There is a lack of possibilities for the correct and complete treatment of pathological entities at visual scale. Cavity preparation depends on a tooth’s shape, position, and the position of adjacent teeth, and cannot always be completely treated (the bacteria and plaque cannot be 100% removed, in some cases) [7]. Sometimes, for different reasons, the possibility exists of cavity infestation remaining or dentin thinning, which can lead to further complication. Correct preparation of cavities is based on systematic procedures (physical and mechanical)—which are not possible for children and for patients with severe anxiety—so the dental restorative material will not remain in the cavity regardless of the nature of the restoration material (modern, based on nanostructures, polymerizable, etc.) [8]. Additionally, between the cement that will fill the cavity and dentin, liners are used, having neo dentin genesis properties. In the final stage, the cavity is filled with restorative materials, one of the main candidates being the glass-ionomer cements (GICs). These are biocompatible, have target compound-release properties, and a coefficient of thermal expansion value close to that of natural teeth, but usually possesses poor mechanical properties.

Some general characteristics of GICs are presented in Table 1.

**Table 1:** Most encountered characteristics of glass ionomer cements.

Polymeric Acids	Glasses	Additives	Water	Physical Requirements According to ISO 9917-1 [9]
poly(acrylic acid) (homopolymer), 2:1 copolymer of acrylic acid/maleic acid	Alumino-silicates (particle size up to 45 μm), zinc silicates, niobium silicates with inclusion of CaF_2_, SrO, SrF_2_, Fe_2_O_3_, etc.	Chelating agents: (+)− tartaric acid, citric acid (5–10%);	11–24%	Luting cement: setting time 2.5–8 min; compressive strength min 70 MPa; acid-soluble As 2 mg/kg; acid-soluble Pb 100 mg/kg; Restorative cement: setting time 2–6 min; compressive strength min 130 MPa; max. 0.05 mm/h; opacity 0.35–0.9; acid erosion acid-soluble As 2 mg/kg; acid-soluble Pb 100 mg/kg

Glass ionomer cements (GICs) represent one of the most used restorative materials in dentistry all over the world. A GIC is generally composed of two phases: a basic glass (i.e., acid-degradable fluoro-aluminosilicate powder), and an acid phase consisting of a polymer liquid [10,11]. With commercial variants available since 1972 [12], GICs have several advantages over other types of materials, including a strong chemical bond with hard dental tissues and clinical metals [13], good adhesion properties in moist environments [14], a prolonged release of fluoride, positive effects on tooth health [15], and lower cytotoxicity when compared with other types of dental restoration materials [16]. Several review articles present GICs and their advantages in practical application as dental restoration materials [11,17,18,19,20]. At the same time, their hydrophilicity allows them to bond to the teeth in the presence of residual fluids [21]. Biocompatibility represents an important factor for any type of material coming in close contact with the human body [22]. This aspect has been covered by several works published over several decades [23,24,25], with classical GICs being identified as less toxic compared with resin-modified or ceramic-reinforced GICs [25]. In order to preserve this important feature, the development of modified GICs should primarily consider the addition of biocompatible materials [26], or, at least, the influence of the added materials on final biocompatibility should be studied [27,28].

One of the main disadvantages of this type of material is related to their poor mechanical and aesthetic properties [11]. As such, their application is partially limited to use as liners [29] and sealing material [30], for periorestoration [31], cementing of glass fiber posts [11], to bond orthodontic brackets to tooth surfaces or cement orthodontic bands [32,33], as a fissure sealant for high-risk caries [34], or even as bulk material in cavities [35]. GICs are also recommended to be used as restoration material for permanent teeth in atraumatic treatments [36].

In practice, depending on their application, different types of GIC are encountered, each type being commercialized for a targeted application [37]:

(a) Luting/bonding cements (Type I)
  - used for cementation, inlays and orthodontic applications;
  - powder/liquid ratio = 1.5/1 … 3.8/1);
  - low setting times;
  - good early resistance to water;
  - radiopaque.
(b) Restorative cements for anterior repairs (Type II *i*), when aesthetic characteristics are important
  - powder/liquid ratio = 3/1 … 6.8/1;
  - corresponding color match;
  - not resistant to water (protection needed);
  - most often radiopaque.
(c) Restorative cements for posterior repairs (Type II *ii*), when aesthetic characteristics are not important
  - powder/liquid ratio = 3/1 … 4/1;
  - low setting times, resistance to water uptake;
  - radiopaque.
(d) Base cements and lining (Type III)
  - powder/liquid ratio = 1.5/1 (for lining), 3/1 … 6.8/1 (for base cements);
  - radiopaque.

The wide application of glass ionomer cements in dentistry are due to a series of intrinsic properties, including their anticarcinogenic character, appropriate biocompatibility and handling, and very good adhesive properties to teeth [38]. These advantages of the GICs are, in some applications (i.e., as permanent fillers), surpassed by their shortcomings, among which their poor mechanical properties are of prime interest [39]. Several approaches were considered for increasing the mechanical properties of GICs, including the addition of reinforcement phases (including metal oxides, such as ZrO_2_, minerals, such as hydroxyapatite, polymeric materials, such as N-vinyl pyrrolidone, fibers, or ceramic additives, among others), or thermo-light polymerization [38,39,40,41]. Each strategy improved the mechanical properties to some extent; however, these results are not yet implemented in clinical applications. Other approaches (currently on the market) involve the development of composites composed of bioactive ionic resin, rubberized resin, and a bioactive ionomer glass, which proved to be effective as restorative materials for primary molars in a one-year clinical study [42].

As can be seen, the GICs’ required characteristics are different depending on the final application. As such, nanotechnology can be used for improving some drawbacks of the material (such as the poor mechanical properties) or enhance other properties (such as the antimicrobial properties).

The present paper aims to review the literature on the application of different types of nanomaterials for the enhancement of GICs’ mechanical and antimicrobial properties, which could lead to several clinical benefits including better physical properties and the prevention of tooth decay. Modification of GICs with metallic nanoparticles (Cu, Ag), metallic and metalloid oxide nanoparticles (TiO_2_, ZnO, MgO, Al_2_O_3_, ZrO_2_, SiO_2_), and apatitic nanomaterials, as well as other nanomaterials or multi-component nanocomposites, is discussed, considering the enhancement of envisaged properties.

## 2. Methodology

For the selection of the studies to be included in the present review, we followed the recommendations of Preferred Reporting Items for Systematic Reviews and Meta-Analyses 2020 (PRISMA) [43]. The research strategy was formulated according to the PICO (Problem, Intervention, Comparison, Outcome) approach (Table 2).

The research was conducted based on the PICO question: “Can inorganic nanomaterials improve the mechanical properties and antimicrobial activity of GICs?” As such, the following inclusion/exclusion criteria were defined:

Inclusion criteria:

- research articles published in the time interval 2011–present, full text;
- articles published or available in English;
- incorporation of nanomaterials (either commercial or obtained in the laboratory);
- randomized clinical trials;
- quantitative and/or qualitative evaluation of mechanical or antimicrobial properties of GIC;
- relevance for the review topic (new information provided).

Exclusion criteria:

- articles published before 2011;
- book chapters or book;
- review or systematic review articles;
- conference paper, note, letter, short survey, erratum or conference review;
- articles published in languages other than English;
- incorporation of exclusively organic materials or carbon nanomaterials in GICs.

The literature search was conducted using the databases SCOPUS (as an exhaustive literature database), PubMed, and Cochrane Library (as specific databases), using “glass ionomer cement” as the primary search term. Further selection of the articles was performed automatically, using the inclusion/exclusion criteria defined above, while inclusion in the present review was decided after a full reading of the manuscript.

## 3. Results and Discussion

**Figure 1:**
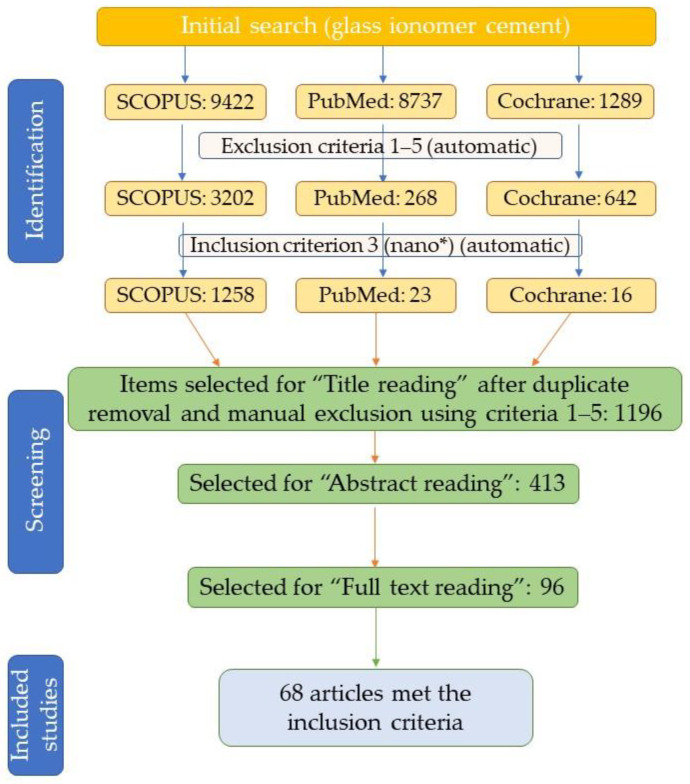
Article selection process flowchart.

After applying the above-stated exclusion and inclusion criteria, as well as title, abstract, and, full text reading, a total of 68 articles were selected for inclusion in the present review (Figure 1), covering the modification of GICs with metallic nanoparticles (Cu, Ag), metallic and metalloid oxide nanoparticles (TiO_2_, ZnO, MgO, Al_2_O_3_, ZrO_2_, SiO_2_), and apatitic nanomaterials, as well as other types of nanomaterials and nanocomposites (nanoclays, multi-component nanocomposites, etc.). To the selected articles, other works were added for providing the necessary context. These articles were retrieved by a “search and find”/manual selection approach using the SCOPUS database (by searching using specific keywords), or were suggested by the reviewers during the peer-review process.

### 3.1. Metallic Nanoparticles in Glass Ionomer Cements

The addition of metallic nanoparticles is usually performed in order to obtain superior antimicrobial properties against specific microorganisms. For example, Ashour et al. [44] evaluated the possibilities to incorporate silver (commercially available) and copper nanoparticles (phytosynthesized using thyme extract) in GIC, with and without the addition of a known antibiotic (metronidazole). Their results showed not only a statistically significant increase in the antimicrobial effect (tested against *Staphylococcus aureus* and *Streptococcus mutans*)—with the silver nanoparticles being more effective compared with the copper nanoparticles, and the composite nanoparticle/antibiotic more effective compared with NPs alone—but also a significant increase in the compressive strength compared with the control (GIC), greater for the nanoparticles alone than for the NPs/antibiotic composite as well as for AgNPs compared with CuNPs. More than that, the long-term efficiency of the CuNP/antibiotic composite seems superior to that of the AgNP/antibiotic composite, suggesting a prolonged effect; in our opinion, this is due to the phytoconstituents acting as capping agents for the NPs. The concentration of NPs in the final composite must be carefully selected, as a higher concentration could alter the bond quality with dentin interaction, as demonstrated by Abed et al. [45].

A similar conclusion was reached using phytosynthesized silver NPs [46]. The use of NPs alone or in combination with a known antibiotic (amoxicillin) led to significantly improved results, both in term of antimicrobial efficiency (compared with GIC, but also with the use of amoxicillin by itself) and in terms of compressive strength (compared with GIC and amoxicillin), with superior results for the application of the composite than for the single use of NPs [46]. Similar results were obtained by Ashour et al. [47] when evaluating the possibilities of incorporating AgNPs phytosynthesized using ginger extract in GIC. The authors observed an increase in both antimicrobial efficiency and compressive strength when surveying the potential of AgNPs and chlorhexidine compared with any of the components used by themselves.

Another metal with potential application as an antimicrobial material in its nanoparticle form is copper. CuNPs showed reduction in colony-forming units upon addition in a 2–4% concentration in GIC in assays against *Streptococcus mutans* and *Streptococcus sanguinis* [48]. The addition of metallic nanoparticles not only induced an increase in the antimicrobial potential of the GIC, but can also increase the mechanical properties of the cements and their dentin-adhesion properties as demonstrated by the examples presented in Table 3.

**Table 3:** Examples of NPs incorporation in GIC (references presented in chronological order) ^1^.

NPs, Ref.	NPs Characteristics	GIC	Experimental Study	Findings
CuNP [44]	Phytosynthesized using *Thymus vulgaris* extract, spherical, 10-25 nm	GC Fuji IX GP ^®^ (type II *ii*)	0.5% NPs, respectively 0.5% NPs + 1.5% metronidazole in GIC, antimicrobial effect tested against *Staphylococcus aureus* and *Streptococcus mutans*; compressive strength measurements	Addition of CuNPs enhanced antimicrobial properties, while not affecting the mechanical properties: IZ = 20/29 mm (*S. aureus),* 19/26 mm (*S. mutans*) after 1 day, 15/19 mm (*S. aureus),* 13/18 mm (*S. mutans*) after 1 month; CS = 44.2/43.9 MPa
AgNP [44]	Commercially available, 20–50 nm			Addition of AgNPs led to superior antimicrobial properties compared with other variants (including CuNPs), while not affecting the mechanical properties: IZ = 24/30 mm (*S. aureus),* 20/27 mm (*S. mutans*) after 1 day, 16/18 mm (*S. aureus),* 14/17 mm (*S. mutans*) after 1 month; CS = 45.9/45.0 MPa
AgNP [45]	Commercially available, under 100 nm	GC Fuji II GP ^®^ (type II *ii*)	0.2, 0.4, respectively 0.6%NPs in GIC, evaluation of the quality of the chemical bond of GIC to primary dentin by FTIR	Concentrations above 0.4% AgNP in GIC altered the bond quality with dentin interaction; addition of AgNPs at low level improves the mechanical properties while maintaining the bond quality
AgNP [46]	Phytosynthesized using *Cupressus macrocarpa* extract, spherical, 13.5–25.8 nm	GC Fuji IX GP ^®^ (type II *ii*)	0.5%NPs, respectively 0.5%NPs + amoxicillin in GIC, antimicrobial effect tested against *Staphylococcus aureus* and *Streptococcus mutans*; compressive strength measurements	Addition of AgNPs showed a synergistic antimicrobial effect with amoxicillin: IZ = 20/30 mm (*S. aureus),* 18/29 mm (*S. mutans*) after 1 day, compared with GIC 9/8 mm, 12/16 mm (*S. aureus),* 11/15 mm (*S. mutans*) after 3 weeks, compared with GIC 0/0 mm; the influence on compressive strength was insignificant: CS = 45.6/45.3 MPa, compared to GIC 44.4 MPa
AgNP [47]	Phytosynthesized using *Zingiber officinale* extract, spherical, 10.5–14.12 nm	GC Fuji IX GP ^®^ (type II *ii*)	0.5% NPs, respectively 0.5%NPs+1% chlorhexidine in GIC, evaluation of antimicrobial activity (against *Staphylococcus aureus*, *Streptococcus mutans*, and *Candida albicans*), CS	The addition of AgNPs and chlorhexidine enhanced antimicrobial efficacy and compressive strength compared with individual components. IZ = 21.3/25.2 (*S. aureus*), 19.4/26.2 (*S. mutans*), 16.3/20.4 (*C. albicans*) at 24 h; IZ = 13.3/18.3 (*S. aureus*), 12.1/19.1 (*S. mutans*), 9.2/16.3 (*C. albicans*) at 3 weeks; CS = 44.7/45.8, compared with 42.4 MPa (control)
AgNP [49]	Commercially available, 20 nm	GC Ortho LC, Fuji ^®^ (type I)	0.15% NPs in GIC, followed by addition of N-acetylcysteine (NAC) at 20% and 2-methacryloyloxyethyl Phosphorylcholine (MPC) 1-3%; evaluation of the bond strength, cytotoxicity, and antimicrobial potential against *Streptococcus mutans*	Cement with AgNP presented strong antibacterial capability, protein-repellent ability, and acceptable biocompatibility. Cell viability 81.3% (day 7), CS = 8.13 MPa (at MPC 2%), suppressed metabolic activity by 59.03% and lactic acid production of biofilms by 70.02%, reduced biofilm CFU by 2 logs, reduced protein adsorption by 76.87%.
AgNP [50]	Chitosan-mediated, hydrodynamic diameter 122 nm	Ketac™ CEM, Easymix,3M (Type I)	10, 30, 50%NPs in GIC, evaluation of mechanical properties and color stability	The addition of AgNPs (10%) induced significant increase in CS = 37 MPa (compared with control 27MPa); the addition of AgNPs also led to significant color change (ΔE = >3.3) and appearance of pores in the cement
AgNP [51]	Commercially available	GC Fuji II GP ^®^ (type II *ii*)	5%NPs in GIC, evaluation of μSBS	Addition of AgNPs increases the bond strength of the restoration: μSBS = 6.96 MPa, compared with control 3.77 MPa
CuNP [48]	Synthesized using ascorbic acid, 10.87 nm	GC Fuji IX GP ^®^ (type II *ii*)	1, 2, 3, 4%NPs in GIC, evaluation of antimicrobial potential against *Streptococcus mutans* and *Streptococcus sanguinis*	Addition of 2–4% CuNPs provided antimicrobial potential to the GIC: HDPFs viability = 68-72% (after 48 h), <10 CFU *S. mutans* (3 and 4%), <20 CFU *S. sanguinis* (4%)
AgNP [52]	Commercially available, 25 nm	Harvard Ionoglas Cem ^®^ (type I)	5%NPs in GIC, evaluation of physico-mechanical properties	Addition of AgNPs significantly increased most of the physico-mechanical parameters: CS~150 MPa, DTS~11 MPa, FS = 29 MPa, H = 90.4 VHN; control CS = 117 MPa, DTS = 7.2 MPa, FS = 27.4 MPa, H = 56.6 VHN;
AgNP [53]	Commercially available	GC Fuji II GP ^®^ (type II *ii*)	0.1%NPs, used as a pretreatment (after the conditioner), evaluation of μSBS	Dentin pretreatment with the nanoparticles after applying the conditioner enhanced the bond strength: μSBS = 3.24 MPa, compared with control 2.17 MPa
AgNP [54]	Commercially available, 20 nm	GC Fuji II GP ^®^ (type II *ii*)	0.1, 0.2%NPs in GIC, evaluation of μSBS, CS, FS, H	GICs with 0.1% and 0.2% AgNPs significantly improved the mechanical properties compared to the unmodified GIC: μSBS = 7.22 MPa, CS = 37.67, FS = 13.03, H = 66.01; control μSBS = 2.14 MPa, CS = 26, FS = 10.92, H = 58.63
AgNP [55]	-	GC Ortho LC, Fuji ^®^ (type I)	1%, 2% NPs in GIC, evaluation of cell viability, H, Ra	Addition of AgNPs led to insignificant differences in cell viability and to significant differences in terms of microhardness and surface roughness compared with control; H = 50.2/33.45 (1/2% NP), control = 54.48 VHN, Ra = 14.76/17.19 μm (1/2% NP), control = 23.45 μm
AgNP [56]	In situ synthesized in poly(acrylic acid) and L-(+)-tartaric acid, 6–11 nm	Fluoro-alumino-silicate ionomer glass powder, poly(acrylic acid) and L-(+)-tartaric acid	Final concentration 0.10–0.50% in GIC, evaluation of CS and antibacterial effect (against *Escherichia coli*)	The addition of 0.5% AgNPs led to significant increase in compressive strength and antimicrobial properties: 32% increase in CS; IZ = 76.1 mm^2^, inhibition of *S. mutans* biofilm
AgNP [57]	Commercially available, <100 nm	GC Fuji IX GP ^®^ (type II *ii*)	1, 3, and 5% NPs in GIC, evaluation of minimum inhibitory concentration and minimum bactericidal concentration (against *S. aureus*), biofilm reduction (against *S. aureus* and *S. mutans*)*,* CS, and H	Addition of silver nanoparticles limits biofilm formation with an insignificant effect on mechanical properties: MIC/MBC = 25/50 μg/mL (*S. aureus*), 25 μg/mL (*S. mutans*); H = 83 (at 1 and 3%), 74 (at 5%), control 85 g/μm^2^. CS = 136/134/132/126 N/mm^2^ (control, 1, 3, 5%NP)
AgNP [58]	Phytosynthesized using *Mangifera indica* leaves, 32 nm	GC Fuji Gold Label Type 9 Glass Ionomer Cement (type I)	3% NPs in GIC, evaluation of H, NPs antimicrobial potential against *E. coli* and *S. aureus*	Incorporation of AgNPs led to improvement of the low wear of GIC and prevented the formation of bacterial colonies. H: 82 (Vickers-VHN), 14.2 (Monsanto-kg/cm^2^), control 54 (Vickers-VHN), 9.5 (Monsanto-kg/cm^2^); IZ = 1.2/1.5 at 8 μg/mL
AgNP [59]	Chemical synthesis, 12 nm	GC Gold Label 1 (type I)	0.1, 0.2% NPs in GIC, evaluation of cytotoxicity (MTT and Trypan Blue assays)	NPs did not affect the cytotoxicity of the GIC (no significant differences being observed).
AgNP [60]	Commercially available, 5–10 nm	GC Ortho LC, Fuji ^®^ (type I)	1, 3, 5, 10, 15% NPs in GIC, evaluation of antimicrobial potential (against *S. mutans*), μSBS	Initially, the incorporation of AgNPs led to significant antibacterial properties, gradually lost with aging time; no antimicrobial effect observed after 8 weeks. A gradual decrease in bond strength was observed with the increasing incorporation of AgNPs, although the results were in the ideal bond strength range: μSBS: 9.58/9.47/9.30/9.07/8.64/7.80 MPa (control, 1/3/ 5/10/15%NP) Addition of AgNPs can decrease the demineralization rate without affecting bond strength

^1^ Abbreviations: NPs—nanoparticles, GIC—glass ionomer cement, IZ—inhibition zone, CS—compressive strength, FTIR—Fourier-transform infrared spectroscopy, DTS—diametral tensile strength, FS—flexural strength, H—microhardness, μSBS—microshear bond strength, HDPFs—human dental pulp fibroblasts, CFU—colony forming units, Ra—surface roughness, ΔE—color variation.

Metallic nanoparticles (the most studied being AgNPs) were found to reinforce the glass ionomer cements, usually by improving their compressive strength; significant improvements of the diametral tensile strength, flexural strength, or microhardness were also observed in several studies (Table 3).

Another aspect (very important for dental materials) is related to the toxicity of the metallic nanoparticles. Known to exert strong cytotoxicity (although the phytosynthesis approach can diminish this character [61]), the nanoparticles seem to exert low [49] or no influence [59] on the glass ionomer cements in which they are included. This aspect is most probably due to the low concentrations in which the NPs are present in the final composites.

Regarding the mechanisms involved in the formation of GIC/metallic nanoparticle composites, the most probable bonding mechanism was proposed to be a mix of micro-mechanical interlocking by surface roughness and chemical interactions through the GIC’s acid copolymers [62]. This would also explain the contribution to the mechanical properties.

### 3.2. Metal and Metalloid Oxide Nanoparticles in Glass Ionomer Cements

Another widely encountered class of antimicrobial materials is represented by the metal oxides (MOx). Several types of metal oxides are known to possess very good antimicrobial efficiency (including, e.g., copper, zinc, silver, or titanium oxides). As it could be expected, these materials were also considered for incorporation in different GICs, most often to increase their antimicrobial potential but also to increase their mechanical properties. Some examples in this area are presented in Table 4.

**Table 4:** Examples of metallic and metalloid oxide nanomaterials incorporation in GIC (references presented in chronological order) ^1^.

NM	NM Characteristics	GIC	Experimental Study	Findings
TiO_2_ [63]	Biosynthesized using *Bacillus subtilis*, 70.17 nm	GC Fuji II GP ^®^ (type II *ii*)	0–10%NM in GIC, evaluation of CS, FS	Addition of TiO_2_ to GIC revealed no observable cytotoxic effect. An increase in the compressive strength and flexural strength was observed for addition of NMs up to 5%. Best results (at 5%NP): CS = 15.51 MPa (control 7.63), FS = 26.39 MPa (control 16.11)
TiO_2_ [51]	Commercially available	GC Fuji II GP ^®^ (type II *ii*)	5% NM in GIC, evaluation of μSBS	TiO_2_ can be incorporated in GIC without compromising the bond strength: μSBS = 4.15 MPa, compared with control 3.77 MPa
ZnO [51]	Commercially available		5% NM in GIC, evaluation of μSBS	Incorporation of ZnO affected the bond strength: μSBS = 2.93 MPa, compared with control 3.77 MPa
TiO_2_ [52]	Commercially available, 21 nm	Harvard Ionoglas Cem ^®^ (type I)	5% NM in GIC, evaluation of physico-mechanical properties	Addition of TiO_2_NMs significantly increased the physico-mechanical parameters: CS = 154.2 MPa, DTS = 13.2 MPa, FS ~28.5 MPa, H ~89 VHN; control CS = 117 MPa, DTS = 7.2 MPa, FS = 27.4 MPa, H = 56.6 VHN
TiO_2_ [53]	Commercially available	GC Fuji II GP ^®^ (type II *ii*)	0.1% NM, used as a pretreatment (after the conditioner), evaluation of μSBS	Dentin pretreatment with the nanoparticles after applying the conditioner enhanced the bond strength: μSBS = 4.81 MPa, compared with control 2.17 MPa
ZnO [53]	Commercially available			Dentin pretreatment with the nanoparticles after applying the conditioner enhanced the bond strength: μSBS = 4.07 MPa, compared with control 2.17 MPa
TiO_2_ [64]	Nanotubes, chemically synthesized, particle size 20 nm, diameter 10 nm	Ketac Molar EasyMix™ (type II *ii*)	3, 5, 7% NM in GIC, evaluation of antimicrobial potential (against *Streptococcus mutans*)	Increased antimicrobial effect with incorporation of 5% NMs: IZ = 8.77/9.06 mm (1 day/7 days) compared with CIG control 8.49/8.41 mm (1 day/7 days). Incorporation of NMs affected *S. mutans* viability and the expression of key genes for bacterial survival and growth. Anticariogenic properties were improved
ZnO [65]	Phytosynthesized using *Syzygium aromaticum* extract	GC Fuji II GP ^®^ (type II *ii*)	50% NM in GIC, evaluation of antimicrobial potential (against *Streptococcus mutans*)	Incorporation of NMs provided antimicrobial activity to the GIC: IZ ~10.5 to 15.5 mm (depending on the *S. mutans* isolate)
TiO_2_ [66]	Nanotubes, chemically synthesized, particle size 20 nm, diameter 10 nm	Ketac Molar EasyMix™ (type II *ii*)	3, 5, 7% NM in GIC, evaluation of CS, FS, μSBS, Ra, WL (after brushing simulation)	Incorporation of NMs improved the mechanical properties and decreased weight loss after surface wear, without affecting adhesiveness to dentin. Best results at 5% NM: CS = 105.23 MPa, FS = 7.41 MPa, μSBS = 5.30 MPa, Ra = 0.3997/0.3851 μm (after/before brushing simulation), WL = 1.4%; control CS = 89.46 MPa, FS = 6.41 MPa, μSBS = 4.76 MPa, Ra = 0.4213/0.3127μm (after/before brushing simulation), WL = 3.8%
MgO [67]	Commercially available	Ketac Molar EasyMix™ (type II *ii*)	1, 2.5, 5, 10% NM in GIC, evaluation of ST, CS, DTS, μSBS	Addition of NMs for up to 2.5% kept the setting time within the requirements of ISO standard, and increased cement strength, without affecting the adhesiveness. Best results at 1% NM: ST ~5.5 min, CS ~240 MPa, DTS ~8 MPa, μSBS (dentin) ~6.2 MPa, μSBS (enamel) ~5.5 MPa
MgO [68]	Commercially available, 20 nm	Ketac Molar EasyMix™ (type II *ii*)	1, 2.5, 5, 10% NM in GIC, evaluation of antibacterial and antibiofilm potential against *Streptococcus mutans* and *S. sobrinus*	Addition of NMs above 2.5% led to the development of materials with antimicrobial activity. Best results at 10% NM: IZ ~8.5/8.8 mm; log10 (CFU/mL) ~6
ZnO [69]	Commercially available	GC Fuji II GP ^®^ (type II *ii*)	1, 5, 10, 15% NM in GIC, evaluation of μSBS, FS, WT, ST	Marginal increase in mechanical properties, no significant differences recorded for any studied parameter
TiO_2_ [70]	Nanotubes, chemically synthesized, particle size 20 nm, diameter 10 nm	Ketac Molar EasyMix™ (type II *ii*)	3, 5, 7% NM in GIC, evaluation of Ra, SH, cytotoxicity	Addition of NMs improved the physico-chemical properties, increased fluoride release, and positively influenced morphology/spreading and extracellular matrix composition. Best results at 5% NM: Ra = 0.49 μm, SH = 118.25 KHN, ECM collagenous and non-collagenous content: 2.94/54.6 μg/well (14 days) control Ra = 0.41 μm, SH = 81.48 KHN, ECM collagenous and non-collagenous content: 2.81/53.3 μg/well (14 days)
TiO_2_ [71]	Commercially available, 21 nm	GC Gold Label 1 (type I)	3%NM in GIC, evaluation of CS, FS, SH	Addition of NMs significantly improved the mechanical properties: FS ~30 MPa, CS ~240 SH ~75 VHN
ZnO [72]	-	GC Fuji II GP ^®^ (type II *ii*)	1 and 2% NM in GIC, evaluation of antibacterial activity (*S. mutans*)	No improvement of antibacterial activity observed
Al_2_O_3_ [73]	Commercially available, <50 nm	3M™ Vitremer™ (type II *ii*)	3.9, 6.1% NM in GIC, evaluation of CL	Addition of NMs improved mechanical properties, without being affected by thermal cycling in artificial saliva; cracks and pores were detected in the modified cement. CL (max. for 3.9%) ~2350 N
ZrO_2_ [73]	Commercially available, <50 nm		4.7, 9.4, 11, 15.8% NM in GIC, evaluation of CL	Addition of NMs improved mechanical properties, without being affected by thermal cycling in artificial saliva; cracks and pores were detected in the modified cement. CL (max. for 4.7 and 9.4%) ~2150 N
TiO_2_ [74]	Commercially available, <25 nm	Dental Shofu FX-II Enhanced Direct Restorative (Type II *i*)	3 and 5% NM in GIC, evaluation of antibacterial activity (*S. mutans*), CS, H, FS, μSBS	Addition of NMs significantly improved mechanical properties and antibacterial activity, without affecting the enamel and dentin adhesion. IZ = 2.11/1.53 mm (control 0.92 mm); CS = 7.3/8.6 MPa, H = 64.2/63.8 VHN, FS = 20.2/21.4 MPa, μSBS dentin = 1.5/0.99, μSBS enamel = 1.96/2.2; control: CS = 5.6 MPa, H = 54.3 VHN, FS = 15.1 MPa, μSBS dentin = 1.32, μSBS enamel = 1.89
SiO_2_ [75]	20–70 nm	Medicem (Type I)	0.01, 0.02, 0.04% NM in GIC, evaluation of bioactivity	Addition of NMs led to the enhancement of the GIC’s bioactivity. Development of a calcium phosphate phase after 1 week immersion in SBF was observed
TiO_2_ [76]	Commercially available, 21 nm	Kavitan ^®^ Plus (Type III)	3, 5, 7% NM in GIC, evaluation of antibacterial activity (*S. mutans*), ST, FT, CS, H, FS, μSBS	Addition of up to 5% NMs improved the mechanical properties without affecting bond strength with dentin or fluoride release. Materials developed possess antimicrobial activity. FT = 1.29/1.33/1.57 MPa/m^2^, CS = 176.27/157.53/92.75 MPa, H = 48.34/36.54/ 28.3 VHN, FS = 23.17/ 19.65/9.12 MPa, ST = 217/204/178 s μSBS = 11.54/10.48/10.14 MPa; control: FT = 0.69 MPa/m^2^, CS = 149.06 MPa, H = 46.3 VHN, FS = 13.57 MPa, ST = 268 s μSBS = 9.46 MPa; BGR = 0.122/0.117/0.112, control = 1.49. Most promising material was proposed to contain 3% NMs

^1^ Abbreviations: NM—nanomaterial, GIC—glass ionomer cement, IZ—inhibition zone, CS—compressive strength, μSBS—microshear bond strength, DTS—diametral tensile strength, FS—flexural strength, H—microhardness, Ra—surface roughness, WL—weight loss variation, ST—setting time, WT—working time, SH—Knoop hardness, ECM—extracellular matrix, VHN—Vickers hardness number, CL—compressive load, SBF—simulated body fluid, FT—fracture toughness, BGR—bacterial growth rate.

Mansour et al. [63] presented the incorporation of anatase- and rutile-phased TiO_2_ nanoparticles obtained by biogenic synthesis (using *Bacillus subtilis*), particle size 70.17 nm, in GIC. They achieved an increase in the compressive strength and flexural strength upon addition of the NPs, as well as observing the non-cytotoxicity of the nanoparticles. What is very important in the cited study is that, as the study was performed over a wide range of NP concentrations (0–10%), the mechanical properties were increased only up to 5% NPs, followed by their decrease. As such, the authors state that there is an optimum NP concentration in GIC; in that specific case, it was found to be 5%. Similar observations were made by de Souza Araújo et al. [64] for TiO_2_ nanotubes, with an optimum concentration also at 5% in antimicrobial assays against *Streptococcus mutans*. The same concentration of TiO_2_ nanotubes was proven to best improve compressive strength and microshear bond strength, as well as to lower weight loss after tooth-brushing simulation, without influencing flexural strength and surface roughness [66,72]. A lower concentration of TiO_2_ in the form of nanoparticles (3%) was previously proposed by Elsaka et al. [76] as the optimum concentration, at which the addition of nanoparticles sufficiently improved the antimicrobial and mechanical properties without compromising other characteristics (such as bond strength and surface microhardness). The optimum nanomaterial concentration is, however, different from material to material. For MgO nanoparticles in GIC, the optimum value was found to be 1% (where the composite presented improvement of the compressive and diametral tensile strength as well as the highest shear bond strength for both enamel and dentin adhesions [67]). However, when evaluated for their antimicrobial potential by the same authors, the MgO nanoparticles seem to be active only above 2.5% concentration in GIC, the best results being obtained at 10% concentration [68] (a very high value, at which the mechanical properties are affected [67]).

When comparing the effect of different nano-fillers on the mechanical properties of modified GIC, Souza et al. [73] observed an improvement of some mechanical properties (compressive strength) when using nano-form Al_2_O_3_ compared with ZrO_2_. However, in both cases, defects (such as pores and cracks) due to the agglomeration of nanoparticles were observed, which could affect the resistance of the materials. In addition, the developed materials were not affected by thermal cycling in artificial saliva.

The antimicrobial effect of the CIG/MOx composites can be explained by different mechanisms. Some oxides (i.e., copper, zinc, or silver oxides, just as their corresponding metallic nanoparticles) act mainly through cellular membrane functionality disruption, generation of reactive oxygen species (ROS), and interference with the intracellular signal transfer pathways [61,77]. For other types of oxides (such as TiO_2_), the antimicrobial mechanisms can be explained by electrostatic interactions between metallic ions and the targeted cell, attachment to the cell membrane, and the ensuing effects on phospholipids [63].

### 3.3. Incorporation of Apatitic Materials in Glass Ionomer Cements

As hydroxyapatite [Ca_10_(PO_4_)_6_(OH)_2_] represents the material closest to the mineral component of teeth [78], it is expected that the materials in the apatite series would be considered for use in dental restoration materials in general, and in particular, for incorporation in GICs. The apatite series includes—besides hydroxyapatite—fluorapatite, chlorapatite, and carbonate-apatite. Apatite materials can be easily synthesized in laboratory conditions, and their properties can be modified to better fit the final application [79]. Recently reviewed [80], the addition of nano-apatitic materials (hydroxyapatite in particular) was found to:

(a) increase the compressive strength by filling the voids in the composite, thus preventing the appearance of defects (such as pores and cracks);
(b) increase flexural strength, due to its porosity;
(c) influence the microhardness of the GIC, usually by increasing it (with superior results for hydroxyapatite in its nanoform compared with microcrystalline material);
(d) improve biocompatibility;
(e) minimize microleakage;
(f) increase fluoride ion release;
(g) increase the antimicrobial properties.

For the purpose of the present review, only works presenting the use of the apatitic materials in their nano form were selected. Table 5 presents some applications of apatitic nanomaterials in GICs as they have emerged from literature data.

**Table 5:** Examples of incorporation of apatitic nanomaterials in GIC (references presented in chronological order) ^1^.

AN	AN Characteristic	GIC	Experimental Study	Findings
HAP [81]	Commercially available	Ketac Molar EasyMix™ (type II *ii*)	5% AM in GIC, evaluation of Ra, H, WL after 60 days of brushing simulation cycles	Addition of AM generated significant changes in the studied parameters: Ra = 1.17 mm (control 0.99), H = 41.19 MPa (control 50.96), WL = −0.00205 g (control 0.00010)
HAP [82]	Commercially available, 20 nm	GC Fuji II GP ^®^ (type II *ii*)	2, 4, 6, 8, 10% AM in GIC, evaluation of ST, CS, H, ML	ST: at concentration above 6%, exceeded imposed limits; at 6%: CS = 158.3 MPa, H = 126.4 MPa, ML = 15.33 (control = 40). No significant changes in the cytotoxicity were observed
HAP [83]	Commercially available	GC Fuji II GP ^®^ (type II *ii*)	1, 2, 5, 7, 10% AM in GIC, evaluation of cytotoxicity	Increased cell viability at 10–99.8% (at 72h, compared with control −91%)
HAP [84]	Obtained by co-precipitation from egg-shells, 39.15 nm	GC Fuji IX GP ^®^ (type II *ii*)	3, 5, 7, 9% AM in GIC, evaluation of H	Higher concentration of AM increased the GIC surface harness. H = 70.21/74.68/ 76.16/79.27 VHN (control – 61.86)
HAP, FHAP [85]	Obtained by microwave-assisted precipitation, different degrees of fluoridation, crystallite size 16.69–22.68 nm	GC Fuji IX GP ^®^ (type II *ii*)	5, 7.5, 10% AM in GIC, evaluation of H	Addition of AM in certain amounts increased microhardness; the difference in fluoridation degrees with the addition of the same mass percentage does not significantly influences the microhardness. Best results: HAP 7.5% (H = 112.17 VHN), 35FHAP 5% (H = 81.23 VHN), 65FHAP 7.5% (H = 80.5 VHN), 95FHAP 5% (H = 81.23 VHN), control 48.94 VHN
HAP [86]	Obtained by co-precipitation, hexagonal, 80–150 nm	GC Fuji I^®^ (type I)	1, 2, 4, 6, 8% AM in GIC at different powder/liquid ratios, evaluation of FS, μSBS	Addition of AM led to the increase in mechanical properties and adhesion potential. Best results at 6% HAP, 3:1 powder/liquid ratio: FS = 30.97 MPa (control 11.65 MPa), μSBS = 0.97 MPa (control 0.39 MPa)
HAP [87]	Commercially available, <200 nm	SDI Riva Self Cure GIC (type I)	1, 3, 5, 8, 10% AM in GIC, evaluation of fluoride release, CS, antibacterial effect (against *S. mutans*)	Addition of HAP increased release up to 8% HAP (0.36 μg/mm^2^), while CS increased for 3–10% HAP (147.12–149.72 MPa), IZ (best results at 8% HAP) ~8.5 mm
HAP [88]	Obtained by co-precipitation, 24 nm	GC Fuji II GP ^®^ (type II *ii*)	5, 8% in GIC, evaluation of CS, DTS, H, ST, WT	Addition of HAP increased mechanical properties: ST = 150/153 s (control 187), WT = 110/108 (control 125), CS ~70/70 (control ~65 MPa), DTS ~9.5/11 (control ~8 MPa), H = 69.3/75.4 (control = 65.3 VHN)
FAP [88]	Obtained by co-precipitation, 30 nm			Addition of FAP increased mechanical properties: ST = 138/135 s (control 187), WT = 98/95 (control 125), CS = ~72/72 (control ~65 MPa), DTS ~11/12 (control ~8 MPa), H = 74.2/77.3 (control = 65.3 VHN)
HAP [89]	Commercially available	GC Fuji II GP ^®^ (type II *ii*)	25% AM in GIC, evaluation of microleakage at enamel and dentin/cementum interface	Microleakage of occlusal margin was significantly lower than that of gingival margin
HAP [90]	Obtained by co-precipitation, 24 nm	GC Fuji II ^®^ (type II *ii*)	5, 8% in GIC, evaluation of CS, DTS, H, ST, WT	Addition of HAP led to an increase in the mechanical properties: ST = 295/215 s (control 340), WT = 215/198 (control 235), CS ~110/112 (control ~105 MPa), DTS ~15/15.5 (control ~12.5 MPa), H = 161.5/168 (control = 158 VHN)
FAP [90]	Obtained by co-precipitation, 30 nm			Addition of FAP led to the increase in the mechanical properties: ST = 275/225 s (control 340), WT = 210/198 (control 235), CS = ~120/120 (control ~105 MPa), DTS ~17.5/19 (control ~12.5 MPa), H = 176.6/201 (control = 158 VHN)
FAP [91]	Obtained by sol-gel, ~100–200 nm	GC Fuji IX GP ^®^ (type II *ii*)	Glass powder/FAP ratio = 20:1, powder/liquid ratio = 3.6/1, evaluation of H, fluoride release, cytotoxicity	Addition of FAP improved surface hardness; H at 7 days = 53.29 kg/mm^2^ (control 46.89); no significant influence on fluoride release and cell proliferation, compared with control, were recorded
HAP [92]	Commercially available, 10–20 nm	Not declared	8% AM in GIC, evaluation of μSBS	Addition of HAP interfered with the bonding ability: 3.28 MPa (control 5.25 MPa); a mixed type of failure was observed for the developed material, while for GIC, a cohesive failure
HAP [93]	Microwave synthesized, calcium deficient, 24 nm	Not declared	5, 10, 15% AM in GIC, evaluation of H, WL, CS, ionic release	The ionic release percentage, weight loss, and compressive strength increased with HAP addition. H ~80/66/58 (control 85 VHN); CS ~102/92/80 (control 68 MPa), increased weight loss and ionic release

^1^ Abbreviations: AM—apatitic nanomaterial, GIC—glass ionomer cement, HAP—hydroxyapatite, Ra—surface roughness, CS—compressive strength, H—microhardness, WL—weight loss variation, ST—setting time, ML—microleakage, VHN—Vickers hardness number, FHAP—fluorhydroxyapatite, FS—flexural strength, μSBS—microshear bond strength, FAP—fluorapatite, DTS—diametral tensile strength, WT—working time.

The addition of commercially available nanohydroxyapatite (HAP) into a restorative material at different concentrations (0–10%) led to the identification of an optimum concentration of 6% HAP, at which the requirements of the ISO 9917-1 standard [9] regarding the setting time are fulfilled, and, at the same time, the physico-mechanical properties (compressive strength, microhardness, and microleakage) are improved [82]. A marginal increase in cell viability was also observed by Golkar et al. [83] when using GIC containing 10% HAP.

The surface hardness of the GIC was found to increase upon the addition of HAP derived from chicken egg shells (significantly for 7 and 9% HAP concentrations) [84], while in other works an optimum concentration was proposed for both HAP and fluorhydroxyapatite (with different degrees of fluoridation) after which the surface hardness starts to decrease [85]; addition of nano-HAP to luting GIC to improve the flexural strength and shear bond strength was also found to have an optimum concentration (6%, at a powder/liquid ratio of 3/1) [86], while the use of a commercial nano-HAP product led to an increase in the mechanical and antimicrobial properties—as well as of the fluoride ion-release capacity—up to an optimum of 8% HAP [87]. Comparing different types of nano-apatitic materials (hydroxyapatite and fluorapatite- FAP), Barandehfard et al. [88,90] obtained superior results in terms of mechanical properties, as well setting and working times, for FAP; this is probably due to the lower solubility rate of FAP (better for 8% FAP compared with 5% FAP). Both apatitic materials presented, however, superior results at both concentrations when compared with the classic GIC used as control [88,90].

As can be seen from Table 5, most of the studied apatitic materials are intended to increase the mechanical properties of GIC. The potential of the apatites (all phosphatic materials) to contribute to these properties is not very surprising, considering that the addition of P_2_O_5_ to the structure of GICs was proven to enhance their mechanical properties [94].

### 3.4. Other Types of Nanomaterials Used in Glass Ionomer Cements

Besides the previously presented materials, the literature presents several studies regarding the use of other types of nanomaterials and nanocomposites for the enhancement of glass ionomer cements’ properties. Table 6 provides some examples in this area.

**Table 6:** Examples of other types of nanomaterials incorporated in GIC (references presented in chronological order) ^1^.

NM	NM Characteristics	GIC	Experimental Study	Findings
BN-TiO_2_ [95]	Chemically synthesized, BN nanosheets (200 nm-1μm) with TiO_2_ grown in situ (20-200 nm); max. thickness of the nanocomposite – 4 nm	China GIC (Chang Shu Shang Chi Dental Materials) (type I)	0.3, 0.7, 1.1, 1.5% NM in GIC, evaluation of H, CS, CoF, So, antibacterial properties (against *Streptococcus mutans*), cytotoxicity (L-929)	The NM served as a reinforcing material for GIC. Data compared with control: H increase: 25.6/77.9/149.65/56.5%; CS increase: 32.8/64.5/ 80.2/52.6%; CoF and So decrease; antibacterial effect increase: 14.5/38.4/67.2/93.4/76.9%; no significant influence on the L-929 cells
Mg_2_SiO_4_ [96]	Sol-gel synthesized, 70–80 nm	GC Fuji II GP ^®^ (type II *ii*)	2, 4, 6% NM in GIC, evaluation of H, CS, FT, fluoride release	Addition of NM led to the improvement of mechanical properties, optimal fluoride release and bioactivity. CS = 850/630/480 MPa (control 350), H = 152/144/131 VHN (control 114), FT = 6.1/4.2/4.3 MPa/m^2^ (control 2.7), slight fluoride-release reduction
ZrO_2_-SiO_2_-HAP [97]	Sol-gel synthesized, 21.62 nm	GC Fuji IX GP ^®^ (type II *ii*)	3, 5, 7, 9% NM in GIC, evaluation of FT, color stability, So, Sp	Addition of NM (especially at 5%) significantly enhanced GIC physico-mechanical properties. FT = 1.16/1.35/1.09/1.05 MPa/m^2^ (control 0.78); ΔE (28 days) = 2.75 (5%, control 3.56), So – 66.46 μg/mm^3^ (control), Sp – 23.64 μg/mm^3^ (control 36.28)
SiO_2_-HAP [98]	Sol-gel synthesized	GC Fuji IX GP ^®^ (type II *ii*)	10% NM in GIC, evaluation of ionic exchange with human enamel and dentin	The addition of NM could provide increased remineralization. Superior levels at ion exchange layer for Sr and Al (enamel), Si, P, Ca (dentin); at 0.1 mm for Ca, Sr (enamel), Al (dentin); at 0.5 mm Si, Sr (enamel), Si (dentin)
SiO_2_-HAP [99]	Sol-gel synthesized, elongated HAP (100–150 nm) covered with SiO_2_ (40 nm)	GC Fuji IX GP ^®^ (type II *ii*)	10% NM in GIC, evaluation of Ra, So, Sp	Addition of NM enhances the GIC physical properties and slightly increased sol-sorption properties. Ra = 0.22 (control 0.22) after 28 days, Sp = 48.38 μg/mm^3^ (control 42.64), So = 63.66 μg/mm^3^ (control 56.65)
rGn-Ag [100]	Synthesized by a chemical method	GC Fuji IX GP ^®^ (type II *ii*)	0.05, 0.1, 0.5, 1, 2%NM in GIC, evaluation of antimicrobial potential, cytotoxicity, FS, H	Addition of 1 and 2% NM significantly decreased the percentage of viable bacteria, without negatively influencing the mechanical properties. FS and H significant increase at 0.1%
Cellulose/ TiO_2_ [101]	Commercial cellulose nanowhiskers, chemically synthesized TiO_2_ (50 nm).	China GIC (Chang Shu Shang Chi Dental Materials) (type I)	2%TiO_2_+1% cellulose in GIC, evaluation of CS, H, enamel μSBS, WR, D, antimicrobial potential (*Candida albicans*), cytotoxicity (L-929)	Addition of NM led to an increase in mechanical properties: CS = 112.7 MPa (control 94.4), no influence on H, enamel μSBS = 14.61 MPa (control 9.69), no significant influence on WR and D; antifungal activity = 92.3% (70 control); slight cytotoxic effect
ZrO_2_-SiO_2_-HAP [102]	Sol-gel synthesized, HAP nanorods—length 114 nm, SiO_2_ 18 nm, ZrO_2_ 39 nm	GC Fuji IX GP ^®^ (type II *ii*)	3, 5, 7, 9%NM in GIC, evaluation of CS, FS, Ra	Incorporation of NM resulted in considerable improvement in the mechanical properties. Best results at 5%: CS = 144.12 MPa (control 117.64), FS = 18.12 MPa (control 14.38). Ra = 0.13/0.15/0.33/0.65 μm (control 0.151)
ZrO_2_-SiO_2_-HAP [103]	Sol-gel synthesized	GC Fuji IX GP ^®^ (type II *ii*)	NM concentration in GIC not disclosed, evaluation of microleakage	Modified GIC had more microleakage than the unmodified cement: 0.96 (control 0.58)
SiO_2_-HAP [104]	Sol-gel synthesized, elongated HAP (100–150 nm) covered with SiO_2_ (~50 nm), different SiO_2_ content (11, 21, 35)	GC Fuji IX GP ^®^ (type II *ii*)	5, 10, 15, 20% NM in GIC, evaluation of H, CS, FS, μSBS	Addition of NM significantly enhanced the mechanical properties of the GIC. Best results for 10% 35SiO_2_-HAP: H = 64.77 VHN (control), CS = 143, 42 MPa (control 119.82), FS = 17.68 MPa (control 11.53), μSBS = 7.85 MPa (control 6.69)
SiO_2_-HAP [105]	Sol-gel synthesized	GC Fuji IX GP ^®^ (type II *ii*)	5% NM in GIC, evaluation of cytotoxicity (MTT assay)	Addition of NM led to an increase in cytotoxicity at 200 mg/mL (cell viability 21.27% at 72 h, compared with control, 57.83%), while no significant differences to control at lower concentrations were observed
ZrO_2_-SiO_2_-HAP [106]	One-pot synthesized SiO_2_-HAP, commercially available ZrO_2_; HAP nanorods: length 140 nm, SiO_2_ 21 nm, ZrO_2_ 40 nm; ZrO_2_ added at different concentration (5, 15, 20, 25% in nanocomposite)	GC Fuji IX GP ^®^ (type II *ii*)	1, 3, 5, 7, 9, 15, 20% NM in GIC, evaluation of H, ΔE	Addition of NM led to the significant improvement of hardness and aesthetic features. Best results at 5% 25ZrO_2_-SiO_2_-HAP: H = 79.38 VHN; ΔE = 4.09 (control 1.99)
Nanoclay [107]	Commercially available, medical grade, 1 nm thickness, 300–600 nm surface dimensions	HiFi glass powder (alumino-silicate glass) and HiFi polyacrylic acid (PAA) powder (Advanced Healthcare Limited)	1, 2, 4% NM in GIC, evaluation of WR, H	No significant influence on WR and H; marginal increase in H at 4% NM
HAP-Ag [108]	HAP commercially available, composite synthesis assisted by γ radiation, 55–65 nm	Transbond XT paste 3M (type I)	1, 5, 10%NM in GIC, evaluation of antimicrobial properties (against *Streptococcus mutans, Lactobacillus acidophilus* and *S. sanguinis*)	Addition of NMs led to a concentration-dependent increase in the mechanical properties: IZ (at 10%) = 8.66/7.66/ 9.66 mm; IZ (at 5%) = 6.33/5.66/7.66 mm; eluted component test: *S. mutans*, significant decrease colony count with concentration increase. *S. sanguinis*, no significant differences between 1 and 5%. Significant reduction at 10%. *L. acidophilus*, no significant differences between 1 and 5%. Biofilm inhibition: *S. mutans*, significant differences between all groups (except 5/10%). *S. sanguinis* and *L. acidophilus*, significant differences between all groups (except between 1/5%, 5/10%)
SiO_2_-HAP [109]	Sol-gel synthesis, elongated HAP (~103 nm), SiO_2_ (~30 nm), different SiO_2_ content (11, 21, 35)	GC Fuji IX GP ^®^ (type II *ii*)	1, 3, 5, 7, 9, 15, 20% NM in GIC, evaluation of H	Addition of NMs led to denser and stronger GIC. Best results at 5% 35SiO_2_-HAP: H = 70.8 VHN (control 40.6)
Nanoclay [110]	Purified nanomer/polymer-grade montmorillonite, (PGV/PGN)	GC Fuji IX GP ^®^ (type II *ii*)	2%NM in GIC, evaluation of CS, DTS, FS, Ef, WT, ST	Addition of nanoclay led to the enhancement of mechanical properties, without negatively influencing the nature of polyacid neutralization. 1-month results: PGV: CS = 122 MPa, DTS = 17 MPa, FS = 24 MPa, Ef = 13 GPa, WT = 4.15 min, ST = 6.55 min; PGN: CS = 130 MPa, DTS = 19 MPa, FS = 28 MPa, Ef = 12 GPa, WT = 4.50 min, ST = 6.50 min; control: CS = 124 MPa, DTS = 16 MPa, FS = 20 MPa, Ef = 11 GPa, WT=4.16 min, ST = 6.35 min
Nanoclay [111]	Polymer-grade montmorillonite	HiFi, Advanced Healthcare (type I)	1, 2, 4%NM in GIC, evaluation of CS, DTS, FS, Ef, WT, ST	Addition of 1/2% NM increased mechanical properties, while 2/4% NM reduced working and setting times. Best 1-month results were recorded at 2%: CS = 134 MPa, DTS = 20 MPa, FS = 43 MPa, Ef = 11 GPa, WT=3.05. Control: CS = 124 MPa, DTS = 18 MPa, FS = 36 MPa, Ef = 14 GPa, WT=3.28 min, ST = 6.30 min
Mg_2_SiO_4_ [112]	Sol-gel synthesized, 36 nm	GC Fuji II GP ^®^ (type II *ii*)	1, 2, 3, 4%NM in GIC, evaluation of CS, FS, DTS	Addition of 1% NM is recommended for applications in which the maximum strength in all three modes of loading is required. CS = 74.4/94.1/106.3/ 38 MPa (control 42.4), FS = 93.7/71.1/31.3/- MPa (control 52,4), DTS = 13/11.7/9.6/- MPa (control 10)
Al_2_O_3_/ ZrO_2_ [113]	Spray pyrolysis, 26 nm	Qingpu NiKang Dental Instrument Manufactory (type I)	Incorporation in GIC alongside HAP and NBG, evaluation of ST, H, YM, W, So, antimicrobial potential (*Pseudomonas, Bacillus*)	Addition of the nanocomposite led to the improvement of mechanical properties, setting time, bioactivity, and antimicrobial activity: ST = 55 s, H = 0.67 MPa, YM = 15.6 GPa, W (after 6 h) = 0.508, initial 0.598 g, So = 15.05%, IZ = 15/14 mm. Control: ST = 110 s, H = 0.43 MPa, YM = 7.77 GPa, W (after 6 h) = 0.478, initial 0.598 g, So = 20.067%.

^1^ Abbreviations: NM—nanomaterial, GIC—glass ionomer cement, BN—boron nitride, CS—compressive strength, H—microhardness, CoF—coefficient of friction, So—solubility, L-929—mouse fibroblasts line, FT—fracture toughness, VHN—Vickers hardness number, HAP—hydroxyapatite, ΔE—color variation; Sp—sorption; Ra—surface roughness, rGn—reduced graphene, μSBS—microshear bond strength, FS—flexural strength, WR—ware resistance, D—dissolution, RWT—Reciprocating wear test, OVW—wear simulator volumetric wear, OWD—wear simulator wear depth, IZ—inhibition zone, DTS—diametral tensile strength, Ef—flexural modulus, WT—working time, ST—setting time, NBG—nano-bioactive glass, YM—Young’s modulus, W—weight.

Ma et al. [95] presented the synthesis of a hexagonal boron nitride/TiO_2_ nanocomposite—obtained by mixing exfoliated hexagonal boron nitride nanosheets and freshly synthesized TiO_2_ nanoparticles—and the incorporation of the nanocomposite in the base powder, followed by the development of modified GIC using different concentrations of nanocomposites (0.3–1.5%). Evaluation of the results led to the proposal that the modified GIC with 1.1% nanocomposite was the material with the highest increase in surface hardness (149.65%) and compressive strength (80.2%) compared with the control (unmodified GIC). The coefficient of friction and solubility also registered the lowest values for this particular concentration, while the antibacterial rate registered the highest increase (93.4%). Neither of the tested concentrations exhibited any significant influence on the GIC’s cytotoxicity. The authors proposed three potential mechanisms by which the nanocomposite enhanced the properties of the GIC: (a) reinforcement of the modified GIC by the evenly distributed ultra-thin composite sheets, which act as conductors of external stress; (b) the action of the rivet-like TiO_2_ which dissipated the external stress, preventing the removal of the nanosheets from the substrate; and (c) at higher concentrations, the TiO_2_ nanoparticles agglomerated in the structure of the modified GIC, becoming structural defects (much more exposed to the action of the external stress). Considering the significant enhancement of the GIC’s properties, the addition of the nanocomposite to a concentration of 1.1% was proposed for the further studies necessary for clinical application.

Another interesting material proposed for incorporation in GICs is forsterite (a member of the olivine and pyroxene mineral groups) [96]. The mineral was synthetically obtained by a sol-gel method and mixed in the GIC’s powder at different concentrations (2, 4, 6%). Evaluating the mechanical properties of the modified GIC, the optimum forsterite concentration was found to be 2%, which was further used for the evaluation of fluoride ion-release tests in artificial saliva. The mineral had a marginal influence on fluoride release, with values lower than that of unmodified GIC; as such, the modified GIC with 2% forsterite was proposed for further studies [96]. When the forsterite concentration was in the range of 1–4%, it was found that 3% increased the compressive strength with 150%, while 1% increase the flexural and the diametral tensile strengths by 80% and 30%, respectively [112].

A three-component composite (ZrO_2_-SiO_2_-HAP) with a particle dimension of 21.62 nm was evaluated by Aldhuwayhi et al. [97] for incorporation in GICs. Considering the fracture toughness results, the 5% composite was selected for further testing, revealing superior color stability, lower water sorption, and higher solubility compared with GIC, which would suggest its possible application in aesthetic restoration. The same composite was previously proven to increase (at the same concentration) the compressive and flexural strength [102], although some slight microleakage was revealed in another study [103].

A similar bicomponent nanomaterial (HAP-SiO_2_) was proposed as a tooth remineralization agent when added to GIC, as proven by the superior levels of P, Ca, Si, Al, and Sr compared with the un-modified GIC, in ion-exchange assays, at different measurement levels [98]; the same material was found in a previous study to increase solubility/sorption capacity without affecting the surface roughness [99], and improve mechanical properties (Vickers hardness, compressive and flexural strength, and shear bond strength), in comparison to conventional GIC [104]. The authors [104] assigned the mechanical-property enhancement to the denser packing of the GIC matrix modified with the optimal nanocomposite concentration. HAP-SiO_2_ incorporated at a 5% concentration in GIC was also evaluated in terms of cytotoxicity by Noorani et al. [105]. The modified GIC was proven to exert a moderate to high cytotoxicity value at 200 mg/mL, but was not significantly different from unmodified GIC at 100 mg/mL and lower concentrations [105]. The authors attributed the increase in cytotoxicity to unreacted polyacrylic acid (PAA) in the composition of the GIC due to some cross-linking of silyl species (nanosilica/glass particles), which limits the number of glass particles available to react with PAA as previously proven [114].

The nanocomposite comprising reduced graphene and silver nanoparticles [100] showed a significant inhibition of *S. mutans* growth in vitro in a composite-concentration-dependent manner. However, the best results for mechanical properties (surface microhardness and flexural strength) were obtained with a 0.1% concentration. Considering all the results, the authors proposed a concentration of 2% for further studies [100].

Nanoclays were also studied as additives in glass ionomer cements. Using polymer-grade and purified montmorillonite, Fareed and Stamboulis [110] proposed their incorporation in GIC at 2%. The authors observed only minor improvement of the GIC’s mechanical properties. Their hypothesis was that the nanoclay does not compromise the nature of polyacid neutralization, thus not affecting the working and setting time while also providing nanoscale reinforcement. The reaction mechanism suggested by the same authors was that the reinforcement is possible through chemisorption and physisorption of PAA on the silicate nanoplates, or even through sodium exchange (in the case of purified nanoclay) and formation of hydrogen bonds [115]. The same authors [111] evaluated different concentrations of polymer-grade montmorillonite addition to GIC. The 1–2% nanoclay addition improved mechanical properties, without negatively influencing the working and setting time.

An Al_2_O_3_/ZrO_2_ nanocomposite synthesized by spray-pyrolysis (particle dimension 26 nm) was also proven to increase the surface hardness and Young’s modulus, while reducing the initial setting time, weight loss, and water solubility, compared with the commercial GIC [113].

## 4. Implications and Future Perspectives

Used for over fifty years, glass ionomer cements are well-established as dental restorative materials with a large area of applications [116]. However, their mechanical properties constitute a barrier for their further development. Nanotechnology can provide instruments for improving those mechanical properties, enhancing the antimicrobial properties, and optimizing their biocompatibility and biomineralizing properties.

The antimicrobial properties of the modified GICs are based on already-established mechanisms, specific to each particular antimicrobial agent. At the same time, improvement of the mechanical properties and the mechanisms involved are still under debate. Whether we are talking about chemical reactions with the base-powder component of the GICs or nanoscale reinforcement of the final cement, an increase in mechanical properties can be achieved using a plethora of nanomaterials. The existence of an ISO standard [9] represents an advantage for rapid development in this area, as it is easier to assess the influence of the nano additives on other important parameters (such as working or setting time, opacity, acid erosion, etc.).

Although there are several examples of laboratory studies which regard the addition of nanomaterials to GIC as having a positive influence, the number of clinical trials is limited. For example, zirconia-improved glass ionomer cements are already marketed and subjected to clinical trials [117]. Nanohydroxyapatite was also evaluated in a controlled trial as a direct pulping agent, used before the application of GIC, demonstrating the production of complete dentinal bridges and an increase in vascularity [118]. Currently, nanohydroxyapatite is under study for the modification of GIC applicable to class V cavities in an in vitro/in vivo study [119], and for the treatment of root caries in geriatric patients [120].

Although the controlled trials represent a very important step towards the development of new products, laboratory research is still necessary in order to improve the properties of GICs. For example, the area of phytosynthesized nanomaterials was explored for addition in GIC. This would overcome the shortcomings of the chemically synthesized nanoparticles in terms in cytotoxicity, as well as increase the antimicrobial properties [61,77]. In future studies, these types of nanoparticles could be incorporated in other types of nanomaterials—such as hydroxyapatite [79]—and used as additives in GICs, as this could increase the mechanical and antimicrobial properties as well as the cements’ biocompatibility.

Further studies are also necessary for developing materials compatible with aesthetic restoration procedures, in order to achieve a color-match with the tooth and maintain color stability. Additionally, all the developed materials should undergo thorough biocompatibility studies in order to ensure a lack of toxicity for the final recipes.

Finally, an important aspect for all types of R&D activities is represented by the possibility of growing in scale. In particular, when speaking of materials that come in intimate contact with the human bodies, the technologies should be reproducible and lead to controlled synthesis of materials.

## 5. Conclusions

Glass ionomer cements, dental materials known for five decades, represent a widely applied solution for problems which require restorative materials. However, their great advantages—including biocompatibility, fluoride release, good thermal expansion coefficient, and excellent teeth bonding properties—are, in some instances, surpassed by their shortcomings, among which their poor mechanical properties are of prime importance.

This review has shown, using data from the published literature, that using different types of nanomaterials can achieve an enhancement of the mechanical and antimicrobial properties; this could provide many clinical benefits, including better physical properties and the prevention of tooth decay. The development of next-generation GICs could bring them to the forefront of dental restoration materials and make them a material of choice.

## Figures and Tables

**Table 2:** Definition of PICO strategy applied for the present work.

P (Problem)	The need for improving the properties of GICs
I (Intervention)	Incorporation of inorganic nanomaterials in GIC
C (Comparison)	Unmodified GIC; GICs modified with other types of materials
O (Outcome)	Improvement of mechanical properties and antimicrobial activity of GICs

## Data Availability

Not applicable.
